# Chemometrics-Assisted UV Spectrophotometric and RP-HPLC Methods for the Simultaneous Determination of Tolperisone Hydrochloride and Diclofenac Sodium in their Combined Pharmaceutical Formulation

**DOI:** 10.3797/scipharm.1306-01

**Published:** 2013-07-14

**Authors:** Nikunj Rameshbhai Gohel, Bhavin Kiritbhai Patel, Vijaykumar Kunvarji Parmar

**Affiliations:** Ramanbhai Patel College of Pharmacy, Charotar University of Science and Technology, CHARUSAT Campus, Changa – 388 421, Ta. Petlad, Dist. Anand, Gujarat, India.

**Keywords:** Tolperisone, Diclofenac, Chemometrics, PCR, PLS, HPLC

## Abstract

Chemometrics-assisted UV spectrophotometric and RP-HPLC methods are presented for the simultaneous determination of tolperisone hydrochloride (TOL) and diclofenac sodium (DIC) from their combined pharmaceutical dosage form. Chemometric methods are based on principal component regression and partial least-square regression models. Two sets of standard mixtures, calibration sets, and validation sets were prepared. Both models were optimized to quantify each drug in the mixture using the information included in the UV absorption spectra of the appropriate solution in the range 241–290 nm with the intervals λ = 1 nm at 50 wavelengths. The optimized models were successfully applied to the simultaneous determination of these drugs in synthetic mixture and pharmaceutical formulation. In addition, an HPLC method was developed using a reversed-phase C18 column at ambient temperature with a mobile phase consisting of methanol:acetonitrile:water (60:30:10 v/v/v), pH-adjusted to 3.0, with UV detection at 275 nm. The methods were validated in terms of linearity, accuracy, precision, sensitivity, specificity, and robustness in the range of 3–30 μg/mL for TOL and 1–10 μg/mL for DIC. The robustness of the HPLC method was tested using an experimental design approach. The developed HPLC method, and the PCR and PLS models were used to determine the amount of TOL and DIC in tablets. The data obtained from the PCR and PLS models were not significantly different from those obtained from the HPLC method at 95% confidence limit.

## Introduction

**Fig. 1:**
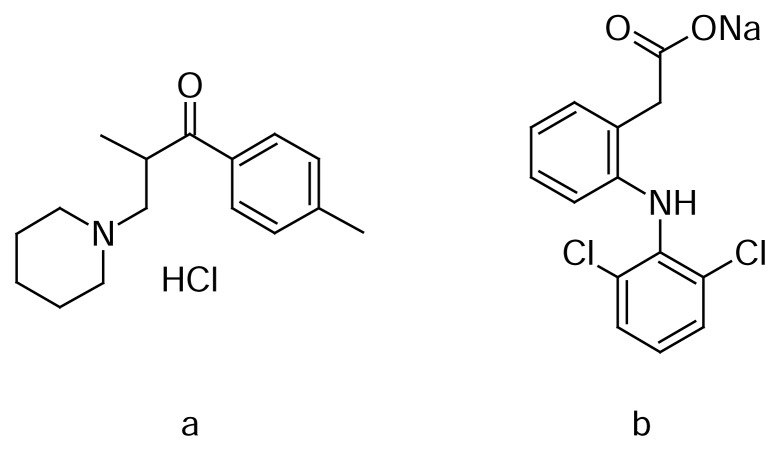
Chemical structure of (a) tolperisone hydrochloride and (b) diclofenac sodium

Tolperisone hydrochloride (TOL), chemically 2-methyl-1-(4-methylphenyl)-3-(1-piperidin-1-yl)propane-1-one, is a piperidine derivative and the structure is shown in Fig. 1[1]. TOL is official in Japanese Pharmacopoeia [2]. It is a centrally acting muscle relaxant which is used in the treatment of different pathological conditions like multiocular sclerosis, myelopathy, encephalomyelitis, spondylosis, spondylarthrosis, cervical and lumbar syndrome, arthrosis of the large joints obliterating atherosclerosis of the extremity vessels, diabetic angiopathy, thromboangitis obliterans, and Reynaud’s syndrome [3]. The literature survey revealed that there are several analytical methods reported for the determination of TOL either individually or in combination with other drugs by spectrophotometric [4], HPTLC [5], and RP-HPLC [6] methods, and are also reported to be in human plasma by GLC [7] and HPLC [8].

Diclofenac sodium (DIC) is chemically the sodium salt of {2-[(2,6-dichlorophenyl)-amino]phenyl}acetic acid and the structure is shown in Fig. 1[1]. It is a non-steroidal anti-inflammatory drug for the treatment of inflammatory conditions such as rheumatoid arthritis, osteoarthritis, and ankylosingspondilytis. DIC is official in Indian Pharmacopoeia [9], British Pharmacopoeia [10], United States Pharmacopoeia [11], and European Pharmacopoeia [12]. The literature survey revealed that several analytical methods have been employed for the quantification of DIC, such as spectrophotometry [13], spectrofluorimetry [14], and chromatography [15]. The combination of TOL and DIC is used for the treatment of adult patients with acute muscle/musculoskeletal spasms. The combination of TOL and DIC is commercially available in tablet dosage form. The literature survey revealed that there are several analytical methods employed for the simultaneous quantification of TOL and DIC such as spectrophotometry [16] and HPTLC [17].

The analysis of TOL and DIC in combination could not be performed by direct UV spectrophotometry without separation due to the overlapping of their UV spectra. The reported UV spectrophotometric methods are based on multicomponent analytical methods viz. simultaneous equation, absorbance ratio, and first derivative methods. These methods are based on the univariate or bivariate calibration methods. In the present work, the chemometrics approach, multivariate calibration methods, is applied for the multicomponent analysis of drug substances with a spectrophotometric method [18–22]. The partial least square (PLS) and principal component regression (PCR) techniques are full-spectrum methods, more powerful than the ones based on a measurement at single or dual wavelength(s), such as direct spectrophotometry, simultaneous equation, or the absorbance ratio method, because the simultaneous inclusion of multiple spectral intensities can greatly improve the precision and applicability of the quantitative spectral analysis of mixtures.

This study aims to introduce an alternative analytical procedure based on the chemometrics-assisted spectrophotometric method for the analysis of TOL and DIC in tablets. An HPLC method was also developed and validated for the simultaneous determination of TOL and DIC. The tablet samples were assayed with the optimized chemometrics-assisted spectrophotometric method and developed HPLC method for comparison. In addition, this work is the first application of multivariate calibration methods, principle component regression (PCR), and partial least square regression (PLS-1), for the determination of TOL and DIC combination in tablets.

## Experimental

### Materials and Reagents

A pharmaceutically pure sample of TOL was procured as a gratis sample from Zydus Cadila Healthcare Ltd., Ahmedabad and DIC was procured as a gratis sample from *Yarrow Chem Products Ltd*, *Mumbai.* Analytical grade reagent orthophosphoric acid (OPA), HPLC grade acetonitrile (ACN), and methanol (AR and HPLC grade) were procured from Loba chemicals, Mumbai, India. A glass distillation assembly from Durga Scientific, Vadodara was used to prepare triple distilled water. The marketed formulation Tolpidol D^®^ containing 150 mg of TOL and 50 mg of DIC was procured from a local market.

### Instrumentation and Software

The Shimadzu UV-1800, a UV-Visible double beam spectrophotometer with a matching pair of 1 cm quartz cuvettes (Shimadzu Corporation, Kyoto, Japan), was used to record the UV spectra of solutions. The spectral band width was 0.5 nm. An integrated HPLC system, LC AT20 from Shimadzu Corporation, Japan was used for the chromatographic separation of TOL and DIC. The HPLC system was comprised of a binary gradient pump and manual sampler, column oven, and a photodiode array detector. PC-installed LC solution software was used to record and integrate the chromatograms. Unscrambler® and MICROSOFT EXCEL were used for PCR and PLS model development and data analysis.

### Chromatographic Conditions

The mobile phase consisted of methanol, acetonitrile, and water in the ratio of 60:30:10 v/v/v and pH-adjusted to 3.0 with orthophosphric acid. A membrane filter of 0.45μm porosity was used to filter and degas the mobile phase. The Enable HPLC Analytical C18 G 120Å (250×4.6 mm, 5μ) was used as a stationary phase. The flow rate was 1.0mL/min and the detector was set at 275 nm. The volume of the sample solution injected was 20 μL. The analysis was carried out at ambient temperature.

### Standard Solutions

#### Preparation of Standard Stock Solutions

TOL powder (100 mg) was accurately weighed and transferred to a 100 mL volumetric flask. It was dissolved and diluted to 100 mL with methanol to obtain a stock solution of TOL with a final concentration of 1 mg/mL.

DIC powder (100 mg) was accurately weighed and transferred to a 100 mL volumetric flask. It was dissolved and diluted to 100 mL with methanol to obtain a stock solution of DIC with a final concentration of 1 mg/mL

#### Preparation of Working Standard Solutions

Standard solution of TOL (10 mL) was transferred to a 100 mL volumetric flask and diluted to 100 mL with methanol to obtain working standard solution of TOL with a final concentration of 100 μg/mL.

Standard solution of DIC (10 mL) was transferred to a 100 mL volumetric flask and diluted to 100 mL with methanol to obtain working standard solution of DIC with a final concentration of 100 μg/mL.

### Calibration Curves for the HPLC Method

Aliquots (0.3, 0.6, 1.2, 1.8, 2.4, and 3.0 mL) of TOL working standard solution (100 μg/mL) and aliquots (0.1, 0.2, 0.4, 0.6, 0.8, and 1.0 mL) of DIC working standard solution (100 μg/mL) were transferred into a series of 10-mL volumetric flasks. The volume was made up to the mark with the mobile phase to yield solutions in the range of 3–30 μg/mL and 1–10 μg/mL of TOL and DIC, respectively. Using a 100 μL syringe, 20 μL volumes of each solution were injected into the liquid chromatograph under the previously mentioned chromatographic conditions. The average peak area of each concentration of TOL and DIC were plotted versus concentrations and the regression equations were computed.

### Calibration of PCR and PLS Methods

#### One Component Calibration

To find the linear dynamic concentration range of each drug, single component calibration was performed. Linear dynamic ranges were studied in the concentration range of 3–30 μg/mL for TOL and 2–12 μg/mL for DIC. Absorbance values were recorded at the λ_max_ of each drug (256 nm for TOL and 282 nm for DIC) in 1-cm quartz cells against methanol as a blank. The linear dynamic range for each compound was determined by least-square linear regression of concentration and the corresponding absorbance.

#### Binary Standard Solutions

Two sets of standard solutions, a calibration set, and a validation set were prepared. Fifteen calibration standards and eight validation standard mixtures were prepared by mixing appropriate volumes of the working standard solutions of TOL and DIC and diluting to volume with methanol. The combination of TOL and DIC are illustrated in Table 1. The absorption spectra of the prepared solutions were measured from 241–290 nm with 1 nm intervals. The absorbance data of the calibration set were then subjected to the Unscrambler^®^ program for the PCR and PLS models. For validation of the PCR and PLS models, the concentrations of TOL and DIC in the validation set were predicted by using the proposed PCR and PLS models.

### Analysis of the Marketed Formulation

Twenty tablets were accurately weighed and finely powdered. Tablet powder equivalent to 100 mg of tolperisone hydrochloride was accurately weighed and transferred to a 100 mL volumetric flask and 50 mL of methanol was added. The mixture was sonicated for 20 min and diluted up to the mark with methanol (solution A) and filtered through a Whatman filter paper no. 41. From this Solution A, 10 ml aliquot was withdrawn into a 100 ml flask and diluted up to the mark with methanol (Solution B). From this Solution B, 1.8 ml aliquot was withdrawn into a 10 ml volumetric flask and diluted up to the mark with the mobile phase for the HPLC method or with methanol for the chemometrics method (Solution C having 18 μg/ml of TOL and 6 μg/ml of DIC) that was used as the final test solution. The peak area of the resulting solution was measured at 275 nm and the concentrations of TOL and DIC were found by fitting the values of peak area in the corresponding linear regression equations of the HPLC method. For chemometric methods, the concentrations of TOL and DIC were found by using a developed PCR and PLS calibration model.

### Validation of the HPLC Method

The HPLC method was validated in compliance with ICH guidelines [23]. The following parameters were validated.

#### Linearity

Working standard solution of the drug was diluted to prepare linearity standard solutions in the concentration range of 3–30 μg/mL and 1–10 μg mL^−1^ of TOL and DIC, respectively. Six sets of such solutions were prepared. Each set was analyzed to plot a calibration curve. Standard deviation (SD), slope, intercept, and correlation coefficient of determination (r^2^) of the calibration curves were calculated to ascertain the linearity of the method.

#### Method Precision (Repeatability)

The precision of the instrument was checked by repeated scanning and measurement of the absorbance of solutions (*n*=5) for TOL (18 μg/ml) and DIC (6 μg/ml) without changing the parameter of the proposed HPLC method. The % RSD was calculated.

#### Intermediate Precision (Reproducibility)

The intraday and interday precision of the proposed method was determined by analyzing the corresponding responses three times on the same day and on three different days over a period of one week for three different concentrations of standard solutions of TOL (12, 18, and 24 μg/ml) and DIC (4, 6, and 8 μg/ml). The result was reported in terms of relative standard deviation (% RSD).

#### Accuracy

The accuracy of the method was determined by calculating the recoveries of TOL and DIC by the standard addition method. Known amounts of standard solutions of TOL and DIC were added at the 80, 100, and 120% level to pre-quantified sample solutions of TOL and DIC (9 μg/ml for TOL and 3 μg/ml for DIC). The amounts of TOL and DIC were estimated by fitting obtained values in the respective regression line equations.

#### Specificity

The specificity of the HPLC method was determined by comparing the chromatogram of the standard and samples of TOL and DIC. Separation of TOL and DIC from the sample solution along with other parameters like retention time (R_t_) and tailing or asymmetrical factor (T) were analyzed.

#### Robustness

The robustness of the HPLC method was tested using the 2^3^ full factorial experimental design. The parameters examined were (A) change in mobile phase composition (B) change in pH of the mobile phase (C) change in the flow rate, and changes in the parameters are mention in Table 2. Factor selection was based on observations during method development and from our own experience. Eight experiments of 2^3^ design to examine the three HPLC factors and their experimental designs are mention in Table 3. All factors were studied at two levels. Responses area and resolution of TOL and DIC were analyzed at each design experiment. The standard solutions of TOL (18 μg/mL) and DIC (6 μg/mL) were measured at each design experiment. The experiment was repeated three times.

#### Limit of Detection and Limit of Quantitation

The limit of detection (LOD) and limit of quantitation (LOQ) were separately determined at a signal-to-noise ratio (S/N) of 3 and 10. The LOD and LOQ were theoretically verified by the equations. LOD = 3.3 σ/m and LOQ = 10σ/m, where, σ is the standard deviation of the intercept and m, the slope of the calibration curve.

## Results and Discussion

### Chemometric Methods

**Fig. 2:**
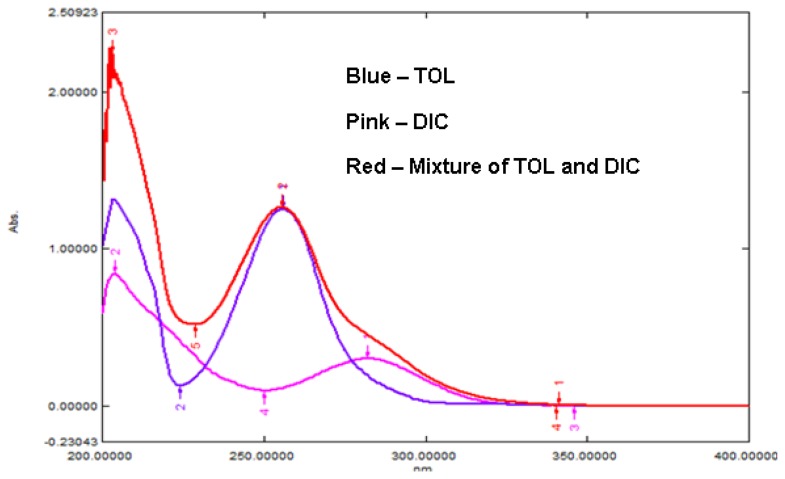
Overlain spectra of TOL, DIC, and mixture

The chemical structures of TOL and DIC are shown in Fig. 1. Fig. 2 shows the UV spectra of these drugs and the mixture of them. As this figure shows, there is clear overlapping between them. The spectral overlapping of these drugs prevents resolution of the mixtures by direct spectrophotometric measurements.

#### Single Component Calibration

To find the linear, dynamic range of each component, calibration graphs were obtained. The absorption spectra were recorded over 200–400 nm against a solvent blank. The linear range for each drug was determined by plotting the absorbance at its *λ*max (TOL, 256 nm and DIC, 282 nm) versus the sample concentration. The calibration curves were linear between 3.0 to 30.0 μg/ml of TOL and 2.0 to 12.0 μg/ml of DIC. The characteristic parameters for the regression equations of individual calibration by absorption of UV spectra are shown in Table 4.

#### Multivariate Methods

The first step in multivariate methods involved constructing the calibration matrix. The wavelength range used was 241 to 290 nm. Fifty spectral points with 1 nm intervals were selected within this range. The compositions of the calibration mixtures were randomly designed in order to collect maximum information from the spectra of these mixtures.

The quality of muiltcomponent analysis is dependent on the wavelength range and spectral mode used. The UV absorption spectra of TOL, DIC, and the mixture at their nominal concentrations are shown in Fig. 2. The calibration set and validation set were randomly prepared with the mixture of TOL and DIC in methanol (Table 1). The UV spectra were observed in the region between 200–400 nm and the absorbances were measured at 50 wavelength points in the region between 241–290 nm with 1 nm intervals.

**Fig. 3:**
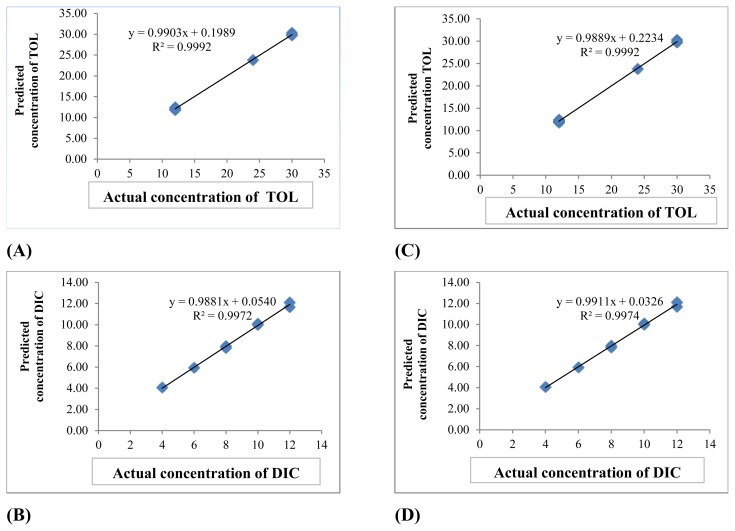
Plot of Predicted vs. Known Concentration for (A) TOL and (B) DIC for PLS method and (C) TOL and (D) DIC for PCR method

The PCR and PLS models were developed by the Unscrambler® program. Model development was performed by using calibration standards. Leave-one-out cross-validation (LOO-CV) was used to validate the PCR and PLS models in model development and obtain optimum latent variables (number of factors) of the model. To select the optimum latent variables (number of factors) in the PLS and PCR algorithms, a cross-validation method, leaving out one sample at a time, was employed using fifteen calibration spectra. The predicted concentrations of the components in each sample were compared with the actual concentrations of the components in each of the validation samples, and the root mean square error of cross validation (RMSECV) was calculated for each method. The RMSECV was used as a diagnostic test for examining the error in the predicted concentrations. The model is the key to achieving the correct quantitation in PLS and PCR calibrations. The parameters of the optimum models are illustrated in Table 5. The resulting models were also validated by prediction of the concentration of analytes in a separate validation set which was not used in model development. The results of prediction and the percentage recoveries are represented in Table 6 and 7. The evaluation of the predictive abilities of the models was performed by plotting the actual known concentrations against the predicted concentrations and the plot of the actual known concentrations against the predicted concentrations are mentioned in Fig. 3. As observed, there was good agreement between the predicted (calculated) and actual concentration of the drugs. The mean recoveries and the relative standard deviations of our proposed methods were computed and are indicated in Table 6 and 7 for TOL and DIC, respectively. Satisfactory correlation coefficient (r^2^) values were obtained for each compound in the validation set by PLS and PCR optimized models indicating good predictive abilities of the models. Another diagnostic test was carried out by plotting the concentration residuals against the predicted concentrations. The residuals appear randomly distributed around zero, indicating adequate model building. The statistical parameters of the validation set are illustrated in Table 8.

### Statistical Analysis

We can define the ability of a calibration in several ways. In this subsection, we calculated the estimations of the standard variation of the chemometric calibrations in the case of the investigated mixtures. The standard error of calibration (SEC) and prediction (SEP) are given by the following expression

$$\text{SEC}(\text{SEP})=\sqrt{\frac{\Sigma_{\text{i}=1}^{\text{N}}{\left({\text{C}}_{\text{i}}^{\text{Added}}-{\text{C}}_{\text{i}}^{\text{Found}}\right)}^{2}}{\text{n}-1}}$$

Here, *Ci*_Added_ represents the added concentration, *Ci*_Found_ denotes the determined concentration, and *n* is the total number of samples. The numerical values of SEC are indicated in Table 5. The SEP of the same mixtures are displayed in Table 8.

The prediction for the residual error sum-of-squares (PRESS) of the calibration step was calculated as:

$$\text{PRESS}=\sum_{\text{i}=1}^{\text{N}}{\left({\text{C}}_{\text{i}}^{\text{Added}}-{\text{C}}_{\text{i}}^{\text{Found}}\right)}^{2}$$

The root mean squares error of cross validation (RMSECV) was calculated for each method as follows:

$$\text{RMSECV}=\sqrt{\frac{\text{PRESS}}{\text{n}}}$$

Where n=number of predicted samples

### Development and Validation of HPLC Method

**Fig. 4:**
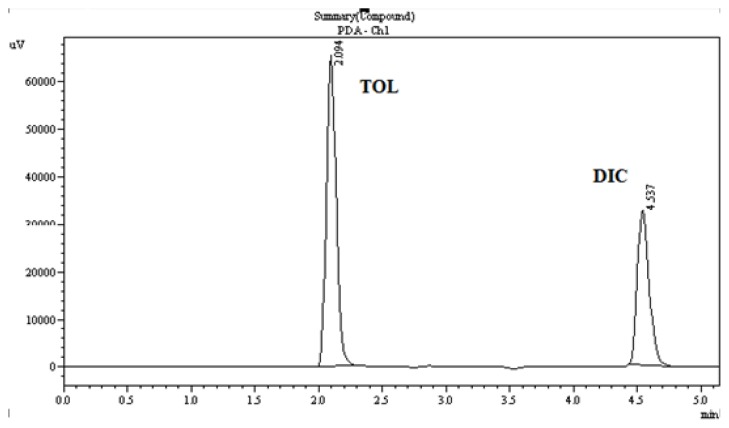
Chromatogram of TOL and DIC

Preliminary studies were performed on a reversed-phase C_18_ column with different mobile phase combinations (methanol:water or acetonitrile:water or methanol:acetonitrile:water) for the optimization of the mobile phase for the HPLC method. A mobile phase consisting of a mixture of methanol:acetonitrile:water (60:30:10 v/v/v), pH-adjusted to 3.0 with *o*-phosphoric acid, was selected as the mobile phase to achieve good sensitivity, good system suitability parameters, and separation of both drugs within 5 min at a flow rate of 1 min/ml. Using a reversed-phase C_18_ column, the retention times for TOL and DIC were observed to be 2.09 and 4.53 min (Fig. 4). The chromatogram at 275 nm shows the complete resolution of all peaks.

The validity of the analytical procedure as well as the resolution between the peaks of interest is ensured by the system suitability test. All critical parameters tested met the acceptance criteria. As shown in the chromatogram, the two analytes are eluted by forming symmetrical single peaks well-separated from each other **(**Fig. 4) and results of the system suitability parameter are illustrated in Table 9.

TOL showed a good correlation coefficient in the concentration range of 3–30 μg/mL (r^2^=0.998), whereas DIC did in the concentration range of 1–10 μg/mL (r^2^=0.998). The linear regression analysis obtained by plotting the peak areas of analytes vs. concentration showed excellent correlation coefficients (correlation coefficients greater than 0.997) and the linearity data are reported in Table 10.

The proposed method afforded high recoveries for TOL and DIC tablets. Results obtained from the recovery studies (Tables 11 and 12), indicate that this assay procedure can be used for the routine quality control analysis of the pharmaceutical dosage form.

The system precision (injection repeatability) is a measure of the method variability that can be expected for a given analyst performing the analysis and was determined by performing six repeats. The %RSD for TOL and DIC response was found to be less than 1.0. The intermediate precision was assessed by analyzing three different concentrations from the calibration linearity on three different days and intra precision was assessed by analyzing three different concentrations from the calibration curve on the same day. The precision studies data are represented in Table 13 and 14 for TOL and DIC, respectively.

**Fig. 5:**
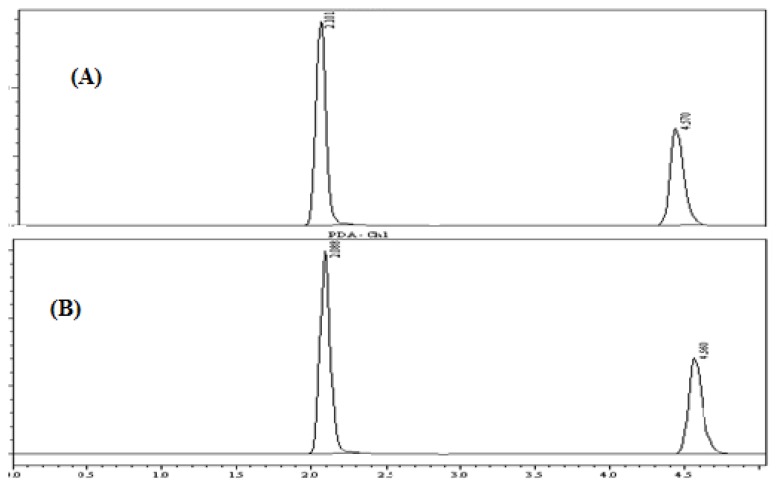
Chromatogram of (A) standard solution and (B) sample solution containing TOL (18 μg/ml) and DIC (6 μg/ml)

The chromatograms were checked for appearance of any extra peaks. It was observed that a single peak for TOL (R_t_ = 2.09) and DIC (R_t_= 4.53) were obtained under optimized conditions showing no interference from common tablet excipients and impurities. Also, the peak area was compared with the standard and the % purity calculated was found to be within limits. These results demonstrate the specificity of the method (Fig. 5).

The limits of quantitation (LOQ) were found to be 0.97 and 0.21 μg/mL for TOL and DIC, respectively. The limits of detection (LOD) were estimated to be 0.32 and 0.07 μg/mL for TOL and DIC, respectively.

At 95% CI, factors are considered as insignificant if the probability (P) value > 0.05. All the independent factors like (A) change in mobile phase composition (B) change in pH of the mobile phase (C) change in flow rate were found to have insignificant effects on all the dependent parameters like % recovery of TOL and DIC and resolution (Table 15). This was concluded from the p values for the above factors found from ANOVA analysis (Table 16). Based on the above statistical analysis, the method was found to be robust.

### Analysis of Market Formulation

The validated chemometrics-assisted UV spectrophotometric and HPLC methods were used in the analysis of the marketed formulation TOLPIDOL D^®^ with a label claim of 150 mg for TOL and 50 mg for DIC per tablet. The results for drug assays show good agreement with the label claims (Table 17).

### Comparison of the PCR and PLS Models with the HPLC Method

In order to compare the results of the proposed PCR and PLS models for the determination of TOL and DIC in tablets, the HPLC method was also employed. The same sample solutions used for the PCR and PLS models were analyzed by the HPLC method. The determination results of PCR, PLS, and HPLC methods are presented in Table 17. The data were expressed in terms of percent labeled amount. The results showed that the average percent labeled amount obtained from the PCR and PLS models were not significantly different from those obtained from the HPLC method with the confidence limit of 95%.

## Conclusion

Principal component regression (PCR) and partial least-square regression (PLS) models were successfully developed for the determination of TOL and DIC in a standard mixture set (validation set) which did not contribute in the calibration set. Similar accuracy was obtained from two multivariate calibration models. A validated HPLC method was also developed for the simultaneous determination of TOL and DIC. The developed HPLC method was found to be sensitive, accurate, precise, and robust. The results of the assay of the commercial formulation obtained from the PCR and PLC models were not significantly different than those obtained from the HPLC method. This implies that the proposed PCR and PLS models are comparable to the HPLC method and can be used for quality control of TOL and DIC in the combined pharmaceutical formulation.

## Figures and Tables

**Tab. 1:** Composition of calibration set and validation set

Standard No.	tolperisone hydrochloride	diclofenac sodium
1c	18	2
2c	3	4
3c	18	4
4c	6	6
5c	18	6
6c	12	6
7c	6	8
8c	24	8
9c	3	10
10c	6	10
11c	18	10
12c	24	10
13c	6	12
14c	18	12
15c	24	12
1v	12	4
2v	30	12
3v	24	6
4v	12	8
5v	30	8
6v	12	10
7v	30	10
8v	12	12

c = solution of calibration set, v = solution of validation set.

**Tab. 2:** The three factors and their levels for 2^3^ experimental design for HPLC

Factors	Levels		
	
	(−)	Nominal (0)	(+)
(A) Change in amount of methanol in mobile phase composition	57 mL	60 mL	63 mL
(B) Change in pH of mobile phase	2.9	3.0	3.1
(C) Change in flow rate	0.95 mL/min	1 mL/min	1.05 mL/min

**Tab. 3:** Eight-experiment 2^3^ design to examine the three HPLC factors

Experiment	Factors		
	
	A	B	C
1	−1	−1	−1
2	1	−1	−1
3	−1	1	−1
4	1	1	−1
5	−1	−1	1
6	1	−1	1
7	−1	1	1
8	1	1	1

**Tab. 4:** Characteristic parameters for the regression equations of individual calibration by absorption of UV spectra

Compound	Regression equation	r2	SD of the slope	SD of the intercept
TOL	y = 0.067x + 0.011	0.999	0.00070	0.01006
DIC	y = 0.051x − 0.0091	0.999	0.00066	0.00253

**Tab. 5:** Statistical parameters of optimum PCR and PLS models for calibration set

Parameters	PCR		PLS	
	
	TOL	DIC	TOL	DIC
Wavelengths Region	241–290 nm	241–290 nm	241–290 nm	241–290 nm
Optimum latent variable	4	6	4	6
RMSECV	0.2112	0.1286	0.2101	0.1257
PRESS	0.4686	0.2320	0.5290	0.2085
SEC	0.1287	0.1944	0.1943	0.1220
Slope	0.9989	1.0067	0.9975	1.0071
Intercept	0.0141	0.0692	0.0438	0.0709
R^2^	0.9995	0.9985	0.9994	0.9986

**Tab. 6:** Results of the prediction set of TOL by PCR and PLS methods

Standard no.	TOL (μg/mL)	Predicted Concentration		% Recovery		Residual	
		
		PCR	PLS	PCR	PLS	PCR	PLS
		
1v	12	12.01	11.99	100.08	99.95	−0.0102	0.0058
2v	30	29.72	29.74	99.08	99.12	0.2756	0.2637
3v	24	23.77	23.80	99.05	99.15	0.2272	0.2048
4v	12	12.42	12.41	103.51	103.43	−0.4207	−0.4119
5v	30	30.28	30.29	100.93	100.97	−0.2794	−0.2909
6v	12	11.78	11.78	98.19	98.16	0.2174	0.2204
7v	30	29.79	29.81	99.30	99.36	0.2112	0.1924
8v	12	12.21	12.20	101.73	101.66	−0.2079	−0.1996

			Mean	100.23	100.22		
			S.D	1.74	1.70		
			%RSD	1.74	1.70		

**Tab. 7:** Results of the prediction set of DIC by PCR and PLS methods

Standard no.	DIC (μg/mL)	Predicted Concentration		% Recovery		Residual	
		
		PCR	PLS	PCR	PLS	PCR	PLS
		
1v	4	4.06	4.06	101.43	101.58	−0.0571	−0.0634
2v	12	11.68	11.66	97.33	97.14	0.3204	0.3431
3v	6	5.92	5.93	98.68	98.82	0.0793	0.0711
4v	8	7.98	7.98	99.75	99.77	0.0201	0.0183
5v	8	7.82	7.80	97.74	97.55	0.1808	0.1960
6v	10	10.08	10.08	100.80	100.84	−0.0795	−0.0842
7v	10	9.99	9.98	99.90	99.84	0.0103	0.0163
8v	12	12.11	12.10	100.93	100.81	−0.1120	−0.0970

			Mean	99.57	99.54		
			S.D	1.52	1.60		
			% RSD	1.52	1.60		

**Tab. 8:** Statistical parameters of optimum PCR and PLS models for validation set

Parameters	PCR		PLS	
	
	TOL	DIC	TOL	DIC
RMSEP	0.2544	0.1433	0.2477	0.1509
PRESS	0.5177	0.1642	0.4912	0.1822
SEP	0.2719	0.1531	0.2649	0.1613
Slope	0.9889	0.9911	0.9903	0.9881
Intercept	0.2234	0.0326	0.1989	0.054
R^2^	0.9992	0.9974	0.9992	0.9972
Bias	0.00166	0.0453	-0.0019	0.05

**Tab. 9:** System suitability test parameters for TOL and DIC by proposed method

System suitability parameter	TOL	DIC
Retention time (min)	2.09	4.53
Resolution factor	–	13.7
Theoretical plates	3026	9147
Tailing factor (asymmetric factor)	1.24	1.36
RSD of area^*^(%)(n=5)	0.71	0.82
RSD of R_T_(%)(n=5)	0.47	0.18

**Tab. 10:** Linear regression data for calibration curve (*n*= 6)

Parameters	TOL	DIC
Linearity range (μg/mL)	3–30	1–10
r^2^ ± SD	0.9984 ± 0.0002	0.9978 ± 0.0008
Slope ± SD	20027.33 ± 128.97	38207.16 ± 183.84
Intercept ± SD	16573.83 ± 1958.75	4971.16 ± 814.41

**Tab. 11:** Results of recovery studies of TOL (*n*=3)

Pre-analysed sample (μg/mL)	Amount of standard added (μg/mL)	Total amount of drug (μg/mL)	Amount of drug recovered (μg/mL ± SD)	% recovery ± SD	% RSD
9	0	9	09.02 ± 0.081	100.26 ± 0.904	0.901
9	3	12	11.86 ± 0.107	98.80 ±0.892	0.902
9	9	18	17.72 ± 0.158	98.45 ±0.880	0.894
9	15	24	23.57 ± 0.063	98.20 ± 0.264	0.268

**Tab. 12:** Results of recovery studies of DIC(*n*=3)

Pre-analysed sample (μg/mL)	Amount of standard added (μg/mL)	Total amount of drug (μg/mL)	Amount of drug recovered (μg/mL ± SD)	% recovery ± SD	% RSD
3	0	3	3.06 ± 0.013	101.96 ± 0.46	0.453
3	1	4	4.07 ± 0.018	101.93 ± 0.44	0.440
3	3	6	6.03 ± 0.015	100.49 ± 0.25	0.254
3	5	8	8.14 ± 0.042	101.82 ± 0.52	0.519

**Tab. 13:** Results of intraday precision and interday precision for determination of TOL (*n*=3)

TOL (μg mL^−1^)	Intra-day precision		Inter-day precision	

	S.D	%RSD	S.D	%RSD
12	1479.98	0.5419	2838.90	1.0395
18	2182.51	0.5859	2337.63	0.6319
24	3360.80	0.6778	1917.86	0.3875

**Tab. 14:** Results of intraday precision and interday precision for determination of DIC (*n*=3)

DIC (μg mL^−1^)	Intra-day precision		Inter-day precision	

	S.D	%RSD	S.D	%RSD
4	1782.70	1.1483	2187.31	1.4089
6	2362.88	1.0569	2637.46	1.1797
8	1098.54	0.3454	3165.07	1.0021

**Tab. 15:** Eight experiment 2^3^ design to examine the three factors (A–C)

Experiments	Factors				Responses		

	A		B	C	% Recovery		Resolution

					TOL	DIC	
1	−1		−1	−1	102.03	100.92	13.95
2	1		−1	−1	99.81	100.04	13.80
3	−1		1	−1	100.75	99.69	13.73
4	1		1	−1	101.62	100.31	13.55
5	-1		−1	1	101.17	101.58	13.74
6	1		−1	1	101.15	101.52	13.11
7	−1		1	1	100.93	100.95	13.51
8	1		1	1	101.37	101.51	13.08

**Responses**	**Effects of Factors**						

	**A**	**B**	**C**	**AB**	**BC**	**CA**	**ABC**

% Recovery of TOL	−0.24	−0.13	0.10	0.88	0.44	−0.13	−0.65
% Recovery of DIC	0.06	−0.40	1.15	0.53	0.19	0.08	0.22
Resolution	−0.34	−0.18	−0.40	0.04	0.18	0.06	0.06

**Tab. 16:** Statistical parameters of experiment obtained by ANOVA

Fac	SS			Df			MS			F			p		
	
	%R		Res	%R		Res	%R		Res	%R		Res	%R		Res
	
	T	D		T	D		T	D		T	D		T	D	
A	0.1	0.0	0.2	1	1	1	0.11	0.01	0.24	0.13	0.08	31.51	0.781	0.829	0.112
B	0.0	0.3	0.1	1	1	1	0.03	0.33	0.07	0.04	3.42	8.76	0.877	0.316	0.207
C	0.0	2.6	0.3	1	1	1	0.02	2.63	0.32	0.02	27.61	42.33	0.902	0.120	0.097
AB	1.6	0.6	0.0	1	1	1	1.56	0.57	0.00	1.82	5.97	0.52	0.406	0.247	0.601
BC	0.4	0.1	0.1	1	1	1	0.39	0.07	0.07	0.45	0.75	8.82	0.623	0.545	0.207
CA	0.0	0.0	0.0	1	1	1	0.04	0.01	0.01	0.04	0.14	0.81	0.872	0.776	0.534
ABC	0.9	0.1	0.0	1	1	1	0.86	0.10	0.01						
Total	3.0	3.7	0.7	7	7	7									

Fac=factors, SS = sum of squares, Df = degree of freedom, MS = mean square, F = F calculated value, p = p value, %R = % recovery, Res = resolution, T = tolperisone hydrochloride, D = diclofenac sodium.

**Tab. 17:** Results of assay in commercial samples

Sample no.	% Amount found					

	PCR		PLS		HPLC	

	TOL	DIC	TOL	DIC	TOL	DIC
1	100.50	98.66	99.00	97.66	99.08	98.10
2	101.27	98.00	100.94	98.33	100.80	98.94
3	98.50	97.50	101.72	100.66	98.40	100.32
4	100.16	99.16	100.44	98.83	99.90	99.05
5	99.88	101.66	98.38	100.33	97.95	98.09

Average	100.06	99.00	100.10	99.16	99.23	98.90

SD	1.01	1.62	1.37	1.29	1.14	0.91
